# To Improve the Initial Inpatient Management of Adolescents Admitted with Severe Anorexia Nervosa: A Narrative Review and a Convenient Protocol

**DOI:** 10.3390/nu14010229

**Published:** 2022-01-05

**Authors:** Stephanie Proulx-Cabana, Marie-Elaine Metras, Danielle Taddeo, Olivier Jamoulle, Jean-Yves Frappier, Chantal Stheneur

**Affiliations:** 1 Pediatrics Department, Division of Adolescent Medicine, Sainte-Justine University Hospital Center, 3175 Côte-Sainte-Catherine Road, Montreal, QC H3T 1C5, Canada; danielle.taddeo.hsj@ssss.gouv.qc.ca (D.T.); olivier.jamoulle.hsj@ssss.gouv.qc.ca (O.J.); jean-yves.frappier.hsj@ssss.gouv.qc.ca (J.-Y.F.); 2 Pharmacy Department, Sainte-Justine University Hospital Center, 3175 Côte-Sainte-Catherine Road, Montreal, QC H3T 1C5, Canada; marie-elaine.metras.hsj@ssss.gouv.qc.ca; 3 CESP, UVSQ, INSERM U 1178, Paris-Saclay University, 94805 Villejuif, France; 4 Clinique FSEF Varennes Jarcy, Fondation Sante des Etudiants de France, 91480 Varennes-Jarcy, France; 5 Simone Veil Health Science Training and Research Unit, Saint-Quentin-en-Yvelines University, 78180 Montigny-le-Bretonneux, France

**Keywords:** Anorexia Nervosa, adolescent, inpatient, medical stabilization, refeeding syndrome

## Abstract

Inadequate nutritional rehabilitation of severely malnourished adolescents with Anorexia Nervosa (AN) increases the risk of medical complications. There is no consensus on best practices for inpatient nutritional rehabilitation and medical stabilization for severe AN. This study aimed to elaborate an admission protocol for adolescents with severe AN based on a comprehensive narrative review of current evidence. A Pubmed search was conducted in July 2017 and updated in August 2020, using the keywords severe AN or eating disorders (ED), management guidelines and adolescent. Relevant references cited in these guidelines were retrieved. A secondary search was conducted using AN or ED and refeeding protocol, refeeding syndrome (RS), hypophosphatemia, hypoglycemia, cardiac monitoring or cardiac complications. Evidence obtained was used to develop the admission protocol. Selective blood tests were proposed during the first three days of nutritional rehabilitation. Higher initial caloric intake is supported by evidence. Continuous nasogastric tube feeding was proposed for patients with a BMI < 12 kg/m2. We monitor hypoglycemia for 72 h. Continuous cardiac monitoring for bradycardia <30 BPM and systematic phosphate supplementation should be considered. Developing protocols is necessary to improve standardization of care. We provide an example of an inpatient admission protocol for adolescents with severe AN.

## 1. Introduction

Anorexia Nervosa (AN) affects up to 0.5% of adolescents [1,2]. It is considered the third most common chronic disease in adolescence and also the psychiatric illness with the highest mortality rate, ranging from 2–8% [1,2]. Part of this high mortality is explained by the multi-systemic stress caused by prolonged fasting, especially on the cardio-vascular system. The risk of death due to medical complications is proportional to the severity of malnutrition at presentation [3]. Hence, providing safe inpatient nutritional rehabilitation to patients presenting with severe AN and medical instability is essential.

Several clinical guidelines have established criteria for hospitalization of adolescents with AN including the Society for Adolescent Health and Medicine (SAHM) [4]. These criteria recommend admission for patients with significant underweight, defined as median BMI ≤ 75% for age and sex, acute medical or psychiatric complications and medical instability of which bradycardia is the most frequent in practice [4].

Although there is a general agreement regarding admission criteria, the initial management of admitted patients with severe malnutrition due to AN is still highly variable with no international consensus on the optimal nutritional rehabilitation for this specific population. In a North American survey, only a minority (37%) of physicians reported using standardized protocols for the initial nutritional rehabilitation phase and large variations were reported for both initial caloric intake and use of nasogastric tube [5]. Even with a same goal of high-calory nutritional rehabilitation, different inpatients protocols from Australia, United States of America and Germany showed major differences, reflecting different practices worldwide [6]. In 2010 and 2011, the Royal College of Psychiatry published the MARSIPAN (Management of Really Sick Patients with Anorexia Nervosa) and Junior MARSIPAN practice guidelines targeting respectively adults and children and adolescent severely ill due to AN, in order to standardize care in the United Kingdom [7,8]. Both constituted the first guidelines designed for the inpatient management of this population, but they were mainly relying on experts’ opinions due to limited evidence. In 2015, the ESCAP (European Society for Child and Adolescent Psychiatry) reviewed the clinical guidelines of four European countries and concluded that research is needed to provide evidence-based recommendations for the management of adolescents with AN [9].

In order to best address the specific population of adolescents with severe AN, this comprehensive literature review was planned with an aim of reviewing current evidence on initial inpatient management of adolescents with severe AN and of establishing an admission protocol applicable to pediatric clinical settings. For each subject integrated in the protocol, we will first present the results of our comprehensive review and then provide the reader with our management practices, based on this review and clinical experience, as included in the inpatient admission protocol.

## 2. Materials and Methods

We performed a comprehensive narrative literature review in July 2017 and repeated the same process again in August 2020 to integrate any new evidence published since.

We first performed a PubMed search by using the keywords: “severe Anorexia Nervosa” or “severe eating disorders (ED)”, management guidelines and adolescent. From the guidelines obtained, specific articles cited as references were retrieved through Google Scholar. After reviewing available guidelines, another Pubmed search was performed to include all other relevant articles (randomized controlled trials, reviews, cohort studies, case-control studies and case reports). The search strategy included the keywords Anorexia Nervosa or eating disorders and each of the following: refeeding protocol, refeeding syndrome, hypophosphatemia, hypoglycemia, cardiac monitoring and cardiac complications.

All relevant guidelines and articles obtained were assessed for applicability to our specific population of adolescents with severe AN admitted for nutritional rehabilitation and medical stabilization.

For this study, we considered patients as severe at admission if there presented with AN and any predisposing factors for high risk of RS [8]:

- Severe malnutrition in adolescents as defined as percent median BMI (%mBMI) ≤ 70% using WHO growth charts or Z-score ≤ −3 SD [4]
- Oral intake <500 kcal/day for ≥3–4 days
- Weight lost >1 kg/week for at least 2 weeks
- Abnormal electrolytes before beginning nutritional rehabilitation

The evidence gathered was analyzed and presented as a comprehensive review. A collaborative admission protocol was created by our medical team and a pharmacist to help medical doctors, general pediatricians and trainees not specialized in the treatment of ED to care for severely malnourished adolescents with AN admitted on a pediatric ward. A list of the studies considered for the development of the protocol is provided in Table 1. The complete protocol is available in Appendix A.

## 3. Results

### 3.1. Nutritional Rehabilitation Protocol

Weight restoration has been associated with better short and long-term outcomes for adolescents with AN, including improvement in cognitive impairment facilitating psychotherapy treatment and reversal of medical complications such as decreased bone density and growth retardation [39]. When dealing with severe malnutrition associated with AN, treatment should be focused on rapid nutritional rehabilitation and weight gain. Benefits of these should however be considered in parallel to the risk of refeeding syndrome (RS), a potentially lethal multi-systemic metabolic reaction triggered by increase in caloric intake in severely malnourished patients [40].

Until recently, conservative initial caloric intake, usually starting at 800 to 1000 kcal/day [39], was recommended by experts as a mean to prevent the development of RS. This approach has been associated with persistent weight loss in the first week of hospitalization [10], without providing evidence of effective reduction of the risk of RS. Articles in the last decade demonstrated that, in patients mildly to moderately malnourished due to AN, risk of RS is similar with higher initial caloric intake [10,11,12,13,14,15]. Furthermore, these studies also showed additional benefits in terms of weight recovery and length of hospital stay [10,11,12,13,14,15], quicker recovery of medical stability [15] and no difference in clinical remission and rehospitalization when compared to lower-caloric nutritional rehabilitation at 1-year [16]. A systematic review by Garber et al. concluded that evidence supported higher initial caloric intake (>1400 kcal/day) for mildly to moderately malnourished patient with AN, but highlighted the lack of evidence regarding those with severe malnutrition due to AN [17]. Due to this lack of evidence, the Junior MARSIPAN still recommend initial caloric intake limited to 5–20 kcal/kg/day [8]. However, literature focused on severe AN has been published since this study. A study in an adult population of 103 patients with severe AN, reported no RS with an initial caloric nutritional rehabilitation plan of 2000 kcal/day with systematic supplementation with phosphate and thiamine [18]. Peebles and al. reported hypophosphatemia requiring supplementation in only 14% of a population of children and adolescents admitted with eating disorders (including AN) refed with mean initial caloric intake of 1466 kcal/day, 84% of which were considered as severely malnourished [19]. None of these patients met the diagnostic criteria for RS [19]. Maginot et al. (2017), reported for 26 severely malnourished adolescents, defined as expected body weight (EBW) of less than 75%, no increased risk of hypophosphatemia, hypomagnesemia or hypokalemia when starting nutritional rehabilitation at higher calory (>1500 kcal/day) [20]. Although there seems to be clear benefits of opting for a higher-calory nutritional rehabilitation, concerns about the impact of the rapid weight restoration on lipid metabolism [21] and the psychological health [41] have been expressed, further highlighting a gap in research needing to be addressed.

**We slowly increase the caloric intake over 5–7 days, depending on intake prior to admission, to reach 2000 kcal/day by the end of the first week of admission**.

### 3.2. Route for Nutritional Rehabilitation

The two main routes for nutritional rehabilitation are meal-based plan and nasogastric tube feeding. There is no current evidence to recommend one over the other [17]. The oral route, constituted of meals and snacks under supervision of specialized ED professionals, is the most common approach in North America. If the patient cannot complete the food, calories can be replaced with nutritional supplements containing 1 to 1.5 kcal/mL [8]. If collaboration to oral feeding is impossible, nasogastric tube feeding should be considered.

Some centers have opted for systematic nasogastric tube feeding in all admitted adolescents mildly to moderately malnourished, limiting the stigmatizing or punitive connotation associated with installing a nasogastric tube after a failure of oral feeding [13]. Their protocol consists of 24-h continuous nasogastric feeding with 1 kcal/mL enteric solution started at 1500–1800 kcal/day. This protocol was associated with better weight gain in the first 2 weeks and reduced length of stay [13]. No patient developed electrolyte disturbances suggestive of RS, provided that 90.3% of patients received prophylactic phosphate supplementation [13]. When nasogastric tube feeding is used, a progressive increase of 200 kcal/day is suggested by the Junior MARSIPAN [8], unless there is a drop in blood phosphate levels. Caloric intake progression should be stopped until phosphate normalizes [8]. The use of nasogastric tube feeding as an adjunct to a meal plan has also been associated with better weight gain during hospitalization in both an adult population and a pediatric population with AN [22,23].

Another potential benefit of 24-h gastric enteral feeding in patients at high risk of RS is that it could limit postprandial insulin peaks related to carbohydrate load [42] and, subsequently, reduce the risk of post-prandial hypoglycemia [43]. Other recommendations to limit the post-prandial insulin surge associated with RS include a diet with less than 40% carbohydrate and with a high phosphate content [43]. Accordingly, the use of 1 kcal/mL enteral formula is preferred in several protocols [13,24], considering that 2 kcal/mL formulas with a greater amount of carbohydrates could increase the risk of RS [8]. Study by Parker et al. (2021) demonstrated that using an enteral formula containing low carbohydrates (28%)/high fat (56%) has been associated with reduced incidence of hypophosphatemia at 1 week of nutritional rehabilitation in adolescents and young adults with AN, although these results should be taken with caution due to the very small sample size (N = 24) and a majority of patient presenting with only mild to moderate malnutrition [25].

**Based on the standard of care of using meal-based plan for admitted patients with AN in our institution, we use this route even with severely malnourished adolescents with AN, with the exception of patients presenting with acute oral refusal, a critical clinical state or BMI Z-score <−3 SD (approximately BMI <12 kg/m2 in patients older than 12 years old). In these patients, we use immediately continuous nasogastric tube feeding to reduce the risk of RS.**

**For digestive tolerance purposes, we take a gradual approach in increasing nasogastric tube feeding rates. We use a 1 kcal/mL enteral solution starting at 20 mL/h for 4 h, with increments of 20 mL/h every 4 h until a maximum rate of 60 mL/h is obtained for the first 24 h. The feeding rate can subsequently be adapted to the weight gain. Such an approach would provide 1440 kcal/day and 1440 mL within the first 24 h to the patient, which is comparable to what would be tolerated by patients with a meal plan. If there is a need to limit fluid intake, a solution of 1.5 kcal/mL could be considered instead.**

### 3.3. Blood Tests

Starvation in AN is associated with depleted total body potassium and phosphate, although serum values are usually initially preserved through metabolic compensation [44]. Refeeding syndrome is a potentially fatal complication caused by intracellular electrolytes shifts due to insulin surges with reintroduction of carbohydrates in a malnourished patient, revealing the absolute electrolytic deficit previously compensated [40]. Although hypomagnesemia and hypokalemia can be observed, hypophosphatemia is the most common and precocious manifestation of RS [26,27,45]. The clinical manifestations of RS are multisystemic, including congestive heart failure, arrhythmias, seizures and coma [46]. The risk of refeeding syndrome is the highest in the first 3 to 7 days of nutritional rehabilitation [28]. In patients hospitalized for AN, the mean incidence of RS in a systematic review was 14% and was more strongly correlated with a % mBMI below 70% than with the initial caloric intake [29,43]. Predisposing risk factors for RS include % mBMI less than 70%, absolute BMI less than 0.4th centile, very low daily caloric intake (<500 kcal/day) for ≥3–4 days, 15% of total weight loss in the last 3 months and electrolyte abnormalities before starting nutritional rehabilitation [8,29,47].

To monitor for RS in newly admitted patients with AN, blood tests are considered part of best practices, but recommendations on frequency are variable. In high-risk patients and those with enteral tube feeding, daily blood samples including electrolytes, phosphorus, calcium and magnesium are recommended for the first 2–5 days of nutritional rehabilitation for early refeeding syndrome surveillance and between days 7 and 10 for late refeeding syndrome [8].

On the other hand, in adolescents with non-severe AN, a study of 3960 blood tests done daily for the first 5 days of nutritional rehabilitation and every other day thereafter in a population of 196 adolescents revealed that only 1.9% were below normal, 0.28% resulted in supplementation and none were associated in a change on nutritional rehabilitation plan [30]. The estimated cost was $1373.73 per patient [30]. Ghaddar et al. also demonstrated in a pediatric population of 99 adolescents with AN, that only 1.61% of performed blood tests revealed abnormal values and 0.85% led to supplementation. There was no difference between the subgroup of patient with a percent median BMI of less than 70% [31]. Once again, average cost was of $1504 per patient, representing an important financial burden [31].

**We perform for all patients admitted for severe AN initial blood tests including complete blood count (CBC), electrolytes, urea, creatinine, blood glucose, ALT, magnesium, phosphorus, calcium and TSH. For any abnormal results in the CBC, urea, creatinine or ALT, we control 48 h later and subsequent control to be based on the attending physician’s judgement. As hypophosphatemia is the first expected manifestation of RS and as daily extended blood tests are not cost-effective, we only monitor electrolytes and phosphate daily the first 3 days after admission, screening for early refeeding syndrome. Bloods tests could then be performed twice a week until the 15th day of hospitalization to cover the late manifestations of refeeding syndrome. If hypophosphatemia of less than 1 mmol/L is noticed, we add a calcium and magnesium assay to the specimen.**

### 3.4. Phosphate Supplementation

During nutritional rehabilitation of an AN patient, a serum phosphate level dropping to 0.9–1 mmol/L or lower can represent the first sign of RS and supplementation should be considered [10,29,46]. In moderate hypophosphatemia (0.35–1 mmol/L), oral route is recommended at a dose of 30–60 mg/kg/day divided into 3–4 doses per day [46]. In severe hypophosphatemia (<0.35 mmol/L), intravenous route under cardiac monitoring is recommended [46].

There is no consensus in the pediatric literature whether systematic phosphate supplementation during the initial nutritional rehabilitation of all patients admitted for AN with severe undernutrition is better than supplementing if serum phosphate decreases to supplementation threshold. In a survey of physicians practicing in the United States and Canada, only 15% prescribed systematic phosphate supplementation for patients with AN as part of inpatient nutritional rehabilitation [5]. An observational study in a pediatric population refed with an initial caloric intake of 1900 kcal/day, only observed mild hypophosphatemia, defined as 0.8–1.1 mmol/L, in 38% of patients, with a median nadir of 4 days. This suggested that, with regular bloods tests, two-thirds of patients would not need phosphate supplements [32]. In an adult population with extreme AN, as defined by ideal body weight (IBW) less than 65%, where systematic prophylactic phosphate supplementation was not used by concerns of diarrhea, only 35% developed hypophosphatemia, with no correlation to initial caloric intake that ranged from 900 kcal/day to 1400 kcal/day [33].

On the other hand, retrospective chart review study conducted by Leitner et al., examined the effects of systematic phosphate supplementation with 1 mmol/kg/day (31 mg/kg/day) for 7 days in admitted adolescents with AN with mild to moderate malnutrition refed at 1800 kcal/day [26]. No patient developed hypophosphatemia while 14.7% had at least one episode of hyperphosphatemia as defined as serum phosphate >1.8 mmol/L within 7 days of starting nutritional rehabilitation. All patients with hyperphosphatemia remained asymptomatic, supporting the opinion of some experts that the benefits of prophylactic phosphate supplementation as a preventive measure of RS probably outweighs the risk of potential side effects [26].

**We start phosphate supplementation for all patients admitted with serum phosphate less than 1 mmol/L or considered at high risk of RS. Due to available formulation in our hospital, an initial dose of 16 mmol sodium phosphate twice a day is used for patient weighing less than 30 kg, whereas a dose of 16 mmol three times a day is used above 30 kg (target dose of approximately 1 mmol/kg/day).**

### 3.5. Sodium and Fluids

Clinical hydration status in patients with AN should be evaluated clinically to establish appropriate fluid requirements [8]. If a patient presents with signs of hypovolemic shock, 0.9% NaCl bolus of 10 mL/kg should be used to avoid precipitating an acute cardiac failure in the context of malnutrition and risk of RS [8]. No specific daily water intake is recommended for the pediatric population. In the adult MARSIPAN and ESPEN guidelines, total daily fluid intake ranging from 20–30 mL/kg/day in the first 72 h of nutritional rehabilitation to 30–35 mL/kg/day is considered appropriate [7,34].

In addition, daily sodium restriction at 1 mmol/kg is recommended in adults to limit the risk of edema [34]. In pediatrics, there is no evidence to support this recommendation.

**We use a total fluid intake of 20–30 mL/kg/day, as suggested in adults. If hemodynamic resuscitation is necessary, we consider NaCl 0.9% 10 mL/kg boluses with close monitoring for cardiac decompensation. We do not limit sodium intakes in pediatric patients. If edema is noted by the nursing team, the medical team will review input/output assessments and blood electrolytes levels (sodium and phosphate) to assess if this could be a sign of RS.**

### 3.6. Hypoglycemia

Hypoglycemia is a rare complication of AN as glucose homeostasis is maintained by compensatory phenomena including increased secretion of GH and cortisol as well as decreased gluconeogenesis due to reduced insulin [48]. In contrast, during nutritional rehabilitation, increased glucose load provokes postprandial insulin surges which can provoke post-prandial hypoglycemia in a pathophysiology similar to the dumping syndrome [49]. Additionally, there is a glycogen depletion in very malnourished patients, leading to dysfunction of the secreted glucagon to counter-regulate this insulin peak [50]. In an adult study of patients with severe AN, with a mean BMI of 13.1 kg/m2, 44% presented mild hypoglycemia (<3.3 mmol/L) and 27% severe hypoglycemia (<2.2 mmol/L). These hypoglycemic episodes lasted for a median period of 8 days and 64% of them were either in the morning or in the post-prandial period [35]. In an adult population with extreme AN, as defined by IBW less than 65%, 38% of adult patients experienced hypoglycemia. This was most likely in those with transaminase levels higher than 3 times the normal and with a greater severity of malnutrition [33].

Literature focusing on hypoglycemia in pediatric patient with AN is sparse. There is no clear frequency for blood glucose monitoring and no clear value for defining significant hypoglycemia. Some authors propose a threshold of <2.5 mmol/L to define hypoglycemia [51] and suggest to check blood glucose in the morning and 2 h after the meals for the first 72h of admission or more frequently if the patient presents symptoms compatible with hypoglycemia [50]. If there is hypoglycemia, continuous enteral feeding could be considered to improve glycemic control [50]. As far as possible, correction of hypoglycemia should be done enterally [8]. However, intravenous correction with a 10% dextrose solution at 2 mL/kg should be used for severe hypoglycemia with impaired consciousness or convulsion. An infusion containing NaCl 0.45% 10% glucose at 5 mL/kg/h has been recommended thereafter to avoid rebound hypoglycemia [8]. In the context that this is not a standard pre-prepared bag of fluids in our institution, and in order to avoid potential errors, we instead use a D10% NaCl 0.9% but at a rate of 3 mL/kg to avoid excessive sodium intake. Glucagon should not be used as it has not been shown to be effective due to low glycogen reserve in malnourished patients with AN [8].

**Considering the lack of strong evidence-based practice, we follow this protocol for hypoglycemia for patients admitted with severe malnutrition due to AN. Glucose levels are monitored before breakfast and 2 h after meals for 72 h. If the patient presents with hypoglycemia defined as <2.5 mmol/L and is asymptomatic, oral correction with a juice and a biscuit will be used as first line of treatment. Blood glucose will be controlled 1 h after snack and if hypoglycemia persists, nasogastric enteral feeding will be started. If the hypoglycemia is severe, <2 mmol/L or symptomatic (with altered state of consciousness or convulsion), intravenous correction will be preferred using D10% at 2 mL/kg, followed by continuous infusion with D10% NaCl 0.9% at 3 mL/kg/h, to avoid rebound hypoglycemia. Continuous nasogastric tube feeding will be started simultaneously with the intravenous infusion aiming at discontinuing intravenous fluids 2 h after starting enteral feeding, if glucose remains >2.5 mmol/L.**

### 3.7. Electrolyte Abnormalities

The presence of hypokalemia, defined as less than 3 mmol/L [10], is usually associated with self-induced vomiting. Whenever possible, oral supplementation should be encouraged [34]. Intravenous supplementation should be considered if there are concerns with oral tolerance or if potassium levels are less than 2.5 mmol/L [8]. The recommended potassium dose for oral repletion is 2–5 mmol/kg/day divided into several doses, not exceeding 2 mmol/kg/dose or 20 mmol per dose [52].

Hypomagnesemia is defined as serum level less than 0.6 mmol/L [24]. There is no clear recommendation regarding supplementation of hypomagnesemia for patients with AN, although some protocols recommend 1–2 tablets of 133 mg of elemental magnesium up to three times a day [11]. The general recommended magnesium dose for hypomagnesaemia supplementation is 20–40 mg/kg/day of elemental magnesium divided into 3 daily doses [52].

**In order to simplify our protocol, we supplement with KCl 20 mEq PO every 8 h, based on an approximate dose of 2 mmol/kg/day for an estimated weight of 30 kg, for a period of 24 h and then reassess. We also use a dose of elemental magnesium of 250 mg PO three times a day for a duration of 3 days if needed.**

### 3.8. Vitamin Supplements

Thiamine is used in the carbohydrate metabolism and thus decreases during nutritional rehabilitation, potentially leading to Wernicke encephalopathy, a neurologic complication associated with thiamine deficiency [53]. In adults with AN, the use of thiamine supplements at a dose of 50 mg orally four times a day for 7–10 days, multi-vitamins and minerals is recommended during nutritional rehabilitation [7]. Pediatric literature is insufficient to support this recommendation. A recent audit of 60 inpatient adolescents admitted with restrictive ED demonstrated no decrease in serum thiamine levels during initial nutritional rehabilitation with the provision of 10 mg of thiamine via a multivitamin [36]. However, as only 11.7% of their sample was considered severely malnourished, they concluded that thiamine supplementation might not be required in mildly to moderately malnourished adolescents with AN [36]. Published recommendations from the American Academy of Pediatrics also support the use of a multi-vitamin in addition to encouraging a varied diet [2]. Nevertheless, their systematic use can be considered reasonable as their potential benefits outweigh the low risk of side effects associated with them [8,47].

**We supplement with a multivitamin during the first 7 days of hospitalization for nutritional rehabilitation. Thiamine supplements are almost always used by our team in patients at high risk of RS at 50 mg PO four times a day for 7 days.**

### 3.9. Cardiac Monitoring

Sinus bradycardia is present in 35–95% of adolescents with AN attributable to an increased vagal tone and decrease in metabolism [54,55]. The risk of developing a junctional escape rhythm or sinus or atrio-ventricular block increases proportionally with the severity of the bradycardia [37,55]. Nevertheless, there is no clear cut-off in the literature to when a continuous heart monitoring should be recommended for bradycardic patients. A review suggested that monitoring should be considered when the heart rate is below 40 beats per minutes (BPM), but this is based on analysis of a few studies documenting mean heart rates in inpatient with anorexia nervosa [37].

In the literature, both prolongation of QTc and increased QT dispersion have been associated with a risk of sudden death [55]. QT dispersion, a marker of ventricular excitability, is defined as the difference between the longest and shortest QT intervals on an EKG and constitutes a significative risk if greater than 100 milliseconds. [37] In a systematic review, Sachs et al. recommended daily EKG if the QTc is more than 470 ms [37]. Continuous cardiac monitoring is recommended if QTc is over 500 ms or if there are identifiable arrhythmias [8,37] and treatable causes should be investigated, including electrolyte abnormalities and medication [8]. The American Psychiatry Association Practice Guide on Adult Eating Disorders suggest considering continuous cardiac monitoring if the weight is less than 70% of the desirable weight [56]. There is no evidence to support this in the pediatric population.

**As continuous cardiac monitoring is only available in the pediatric intensive care unit in our center and that evidence to support a cut-off of 40 BPM is sparse, we have maintained our previously established cut-off of 30 BPM for continuous cardiac monitoring.**

**We perform systematic EKG in all adolescents admitted for AN. If the QTc is greater than 470 ms, we repeat daily EKG until normalization. If the QTc is greater than 500 ms, we initiate continuous cardiac monitoring.**

### 3.10. Echocardiography

Cardiac ultrasonography can reveal structural cardiac complications associated with AN. Although myocardial atrophy is frequently observed, it is usually not associated with significant cardiac dysfunction [37,55]. Prolapse of the mitral valve is common, reported in 33–66% of patients, and manifest as chest pain with palpitations, a mid-systolic click or a systolic murmur [55]. Finally, 22–35% of adolescents with AN have a degree of pericardial effusion, the vast majority remaining asymptomatic [37]. All these complications are reversible with nutritional rehabilitation [38]. This suggests that a routine cardiac ultrasound screening for all patients admitted with severe AN is not indicated as rarely will it affect the natural trajectory of these complications or the patient’s management plan. Cardiac ultrasonography should be considered in the presence of cardiac symptomatology, abnormal cardiac examination or EKG abnormalities [37,57]. If any structural abnormalities are identified in an initial cardiac ultrasound, a cardiac ultrasound should be repeated 30 to 60 days after [37].

**Cardiac ultrasound is left to the discretion of the treating physician.**

## 4. Perspectives

The admission protocol constituted of our recommendations is available as an appendix to this article. This protocol has been developed based on the aforementioned comprehensive review of the literature and with the collaboration of different members of our team. By covering nursing plan, blood tests, nutrition plan, management of hypoglycemia and supplements, we believe that this protocol details all aspects of the initial management of adolescents admitted with severe malnutrition due to AN and can be valuable for all professionals dealing with stabilization of young people during the initial nutritional rehabilitation period.

There are limitations to our study. As previously mentioned, due to a lack of relevant evidence on multiple subjects, especially in the adolescent’s population with severe AN, many recommendations that were integrated as part of this study had to be based on adult literature, studies including mostly mildly to moderately malnourished adolescents with AN and expert’s consensus. Another limitation of this study is that, although we have tried to make this as comprehensive as possible, we have not used a systematic review framework and there is a possibility that we have not correctly collected all possible evidence available in the literature. A systematic review about the initial inpatient stabilization of adolescents with severe AN would provide a more thorough insight, completing this narrative review. However, due to severe malnutrition being used as an exclusion criterion in many studies about nutritional rehabilitation, this might make formulating conclusions for the pediatric population with severe AN quite difficult for each specific subject addressed in our study. There is a clear need for more research in the severe AN population in order to provide clear evidence-based practices, especially for initial caloric intakes, hypoglycemia management and criteria for continuous cardiac monitoring.

## 5. Conclusions

Managing the initial nutritional rehabilitation of adolescents admitted with severe AN remains complicated even for experienced physicians. The absence of clear guidelines addressing all aspects of this crucial period makes it even harder for general pediatrician caring for these young people outside of a specialized eating disorders unit. Our team’s comprehensive review of the current literature has been used to create an example of inpatient admission protocol for adolescents with severe AN based on current evidence. Due to lack of strong evidence, relying on expert consensus was inevitable at this time for some aspects of the management but provide focus on current gaps in knowledge that need to be address by research for this specific population.

## Figures and Tables

**Table 1:** Studies included in the conception of the protocol.

Study	Year of Publication	Country	Study Type	Population							Conclusion
				Number	Age	Sex	BMI	ED Subtype	Study Setting	Length of Observation Period	
Davies et al. [3]	2017	United Kingdom	Cohort study	65	Median age 24	N.A.	<13 kg/m^2^	AN	2 multidisciplinary eating disorder services	90 days	Starting at low-calory intakes (20–30 kcal/kg), 6.5% patients developed mild hypophosphatemia and none developed RS
Garber et al. [10]	2012	United States of America	Cohort study	35	13.1–20.5	97% female	80.1%mBMI (mean)	AN	Terciary care children’s hospital	16.7 days (mean)	83% of patients initially lost weight at a 1200 kcal/day diet.
Golden et al. [11]	2013	United States of America	Retrospective cohort study	310 (88 LCR/ 222 HCR)	10-21	88.4% female	78.5% mBMI	AN	Terciary care children’s hospital	First admission	Length of stay significantly shorter in HCR by 3 days. No difference in between groups in terms of hypophosphatemia, hypomagnesemia and hypokalemia, including in a subanalysis of severely malnourished group.
Madden et al. [12]	2015	Australia	Cohort study	78	12–18	94.87% female	78.37% EBW	AN	Two specialist pediatric eating disorder services	2.5 week admission	Immediate weight gain No refeeding syndrome
Agostino, Erdstein, & Di Meglio [13]	2013	Canada	Non-randomised controlled study	165 (31 HCR/134 LCR)	10–18	94–96% female	82–85% IBW (mean)	Restrictive eating disorder	Terciary Pediatric Hospital	First 2 weeks of admission	Reduced length of stay, better weight gain in HCR nasogastric feeding group. No complication including no RS with use of phosphate supplements
Parker et al. [14]	2016	Australia	Retrospective cohort study	162	14–19	91% female	80.1% BMI	Restrictive eating disorder	Adolescent ward	Admission for nutritional rehabilitation	HCR protocol with phosphate supplements provided rapid weight gain and no increase incidence of RS.
Garber et al. [15]	2021	United States of America	RCT	111 (60 HCR/51 LCR)	12-24	91% female	≥60% mBMI	AN and AAN	2 tertiary care eating disorder programs	Time to medical stability	Medical stability 3 days faster and heart rate restoration 4 days in HCR group. Shorter length of stay by 4 days in HCR. Similar electrolyte disturbances in both groups.
Golden et al. [16]	2021	United States of America	RCT	111 (60 HCR/51 LCR)	12–24	N.A.	≥60% mBMI	AN and AAN	2 tertiary care eating disorder programs	12 months	No difference in clinical remission and medical rehospitalization at 1-year between HCR and LCR
Garber et al. [17]	2016	United States of America	Systematic review	26 studies	N.A.	N.A.	N.A.	AN	N.A.	N.A.	LCR is too conservative in mildly-moderately malnourished patient Meal-based approaches or combined nasogastric+meals can administer HCR. HCR has not been associated with an increased risk of RS. There is insufficient evidence in severely malnourished patients.
Koerner et al. [18]	2020	Germany	Retrospective chart review	103	18–47	Female	<13 kg/m^2^	AN-R, AN hyperactivity subtype and AAN	Unit for extremely underweight patients with AN	Stay >4 weeks	HCR for nutritional for severe patients with AN did not increase the risk of RS.
Peebles et al. [19]	2017	United States of America	Retrospective chart review	215	5.8–23.2	88% female	86% mBMI (mean)	64% AN, 18% AAN, 6% BN, 5% PD, 4% ARFID and 3% UFED	Pediatric hospital	First-time admission for nutritional rehabilitation	84.2% of their sample considered severely malnourished. Only 14% needed phosphate supplementation. No RS. Only 3.8% readmitted within 30 days.
Maginot et al. [20]	2017	United States of America	Retrospective chart review	87 (21 LCR/66 HCR)	8–20	81% female (LCR) and 84.9% HCR	LCR: 78.7% EBW HCR: 81.2%EBW	AN-R (66.7%), AN-B/P (16.1%), ARFID (11.5%) and UFED (5.7%)	Children’s inpatient medical stabilization unit	Medical stabilization admission	Secondary analysis on severely malnourished (<75% EBW) (N = 26). No increase risk of hypophosphatemia, hypogmagnesemia or hypokalemia in the first 72h with HCR. LCR more likely to be readmitted than HCR (40% vs. 6%).
Tam et al. [21]	2021	Germany	Non-randomised controlled study	76 (39 underweight patient with acute AN/ 37 control	12–28	Female	T1-15.0 kg/m^2^ T2-19.5 kg/m^2^	AN	Intensive inpatient treatment of a specialized eating disorder program	Median time to weight restoration 85 days (35–140)	Dysruption of plasma lipodome after short-term weight restoration similar to those in obesity and metabolic syndrome.
Rigaud et al. [22]	2007	France	RCT	81 (41 nasogastric feeding/ 40 control)	18–28	97% female	12.1–12.8 kg/m^2^(mean)	AN-B/P and AN-R	Inpatient nutrition unit	1 year follow-up	Weight gain 39% higher with nasogastric feeding.
Robb et al. [23]	2002	United States	Retrospective chart review	100 (48 oral refeeding/ 52 nocturnal nasogastric feeding	15 (mean)	Female	15.5–16 kg/m^2^ (mean)	AN	Academic pediatric hospital	Hospital admission	Greater and more rapid weight gain. No difference in length of stay
O’Connor et al. [24]	2016	United Kingdom	RCT	36 (18 LCR 500 kcal/day group vs 18 “HCR” 1200 kcal/day	10–16	94% female	<78% mBMI	AN	6 United Kingdom Hospitals	10 days of nutritional rehabilitation	Group at 1200 kcal/day had greater weight gain. Hypophosphatemia was associated with initial mBMI and electrolyte abnormalities before treatment but not caloric intakes.
Parker et al. [25]	2021	Australia	RCT	24 (14 low carbohydrate/high fat formula/ 100 standard formula	15–25	Female	77–79% mBMI (mean)	AN	2 hospital with inpatient eating disorder services	1 week	Lower rate of hypophosphatemia in treatment group
Leitner, Burstein, & Agostino [26]	2015	Canada	Retrospective chart review	75 admissions	<18	95% female	83.5% mBMI (mean)	AN or other restrictive eating disorder	Tertiary pediatric hospital	First 7 days of nutritional rehabilitation	With systematic phosphate supplementation, no episodes of refeeding hypophosphatemie and 14.7% mild asymptomatic hyperphosphatemia.
Brown et al. [27]	2015	United States of America	Retrospective case-control study	123 (69 AN-R/54 AN-B/P)	>17	Female	62.6%IBW (mean)	Severe AN-R and AN-B/P	Specialized medical stabilization unit for severely-comprised eating disorder patient.	Admission for medical stabilization	Prevalence of hypophosphatemia was 33.3%. Only identified risk factor was higher hemoglobin. Protective factors higher BMI, higher serum potassium and prealbumin.
Friedli et al. [28]	2016	N.A.	Systematic review	45 studies on RS (16 studies specifically on AN)	All ages	N.A.	N.A	AN and other medical conditions leading to malnutrition	N.A	N.A.	In studies reporting timing, most reported within 72 h of starting nutritional rehabilitaiton. Risks factors for refeeding syndrome include malnutrition, low electrolyte concentration and history of alcohol abuse.
O’Connor & Nicholls [29]	2013	United Kingdom	Systematic review	17 articles for a total of 1039 subjects	10–20	N.A.	78% mBMI (mean)	AN	N.A	N.A	Average incidence of refeeding hypophosphatemia 14%, Significant correlation between %mBMI and post-refeeding phosphate.
Ridout et al. [30]	2016	United States of America	Retrospective chart review	196 patients encounters	15.9 (mean)	87% female	89% mBMI	AN, BN or UFED	Adolescent Medicine Service at a Children hospital	Bloods tests daily for 5 days then every other day until discharge.	No cases of RS. Total of 3960 laboratories obtained of which 1.9% were below normal, 0.05% critical values et 0.28% led to supplementation. Total laboratory costs were $269,250.85
Ghaddar et al. [31]	2019	Canada	Retrospective chart review	99 admissions	<18	97% female	15.3 kg/m^2^ (mean)	AN-R or AN-B/P	Pediatric tertiary center	All blood tests performed daily within the first week of nutritional rehabilitation.	1289 laboratory tests performed of which 1.5% revealed abnormal values and 0.85% led to supplementation. Total cost 148,926.80 CAD$
Whitelawet al. [32]	2010	Australia	Retrospective chart review	46 admissions (92% HCR ≥1900 kcal/day)	12–18	N.A.	72.9% IBW (mean)	AN	Tertiary pediatric hospital	Initial 2 weeks	Only 38% developed mild hypophosphatemia thus supporting monitoring instead of prophylactic phosphate. Patients with %IBM < 68% were at increased risk of hypophosphatemia.
Gibson et al. [33]	2020	United States of America	Retrospective chart review	281 (62% AN-R)	91% female	18–66	<65% IBW	AN-R and AN-B/P	Sever anorexia specialized adult unit.	Admission for medial stabilization	In this extreme AN group, with average initial caloric intakes of 1431 kcal/day, 38% developed hypoglycemia, 35% refeeding hypophosphatemia, 33% edema. Highly elevated LFTs predicted hypoglycemia and low BMI predicted hypophosphatemia.
Hofer et al. [34]	2014	Switzerland	Retrospective chart review	86 admissions	93% female	>16	74.4% had <70%IBW	AN	Inpatient unit.	30 days inpatient and 3 months	Protocol for LCR starting at 10 kcal/kg and fluid restriction of 20–30 mL/kg. During nutritional rehabilitation, supplement in potassium (47.7%), in phosphate(32.6%) and in magnesium (40.7%). Pre-tibial edema was present in 4.7%.
Gaudiani et al. [35]	2012	United States of America	Retrospective chart review	25 consecutive admissions for severe AN	88% female	18–46	62% IBW (mean)	AN	Specialized adult unit for medical stabilization of severe AN	Medical stabilization admission	With a mean initial caloric intake of 990 kcal/day, 44% had mild hypoglycemia and 12% severe hypoglycemia. Glucose was the lowest early in the morning and post-prandial. Median time to resolution hypoglycemia was 8 days. 76% had abnormal LFTs. 45% developed hypophosphatemia with a mean time of 3.4.
Parker et al. [36]	2020	Australia	Retrospective chart review	60 admissions (62% AN-R, 23% AN-B/P, 10% ARFID and 5% AAN)	88% female	17.2 (mean)	80.4% mBMI (mean)	AN-R, AN-B/P, AAN and ARFID	Tertiary hospital	Weekly bloods during admission	With an average initial intake of 2482 kcal/day and multivitamin containing 10 mg of thiamine, no patient had blood thiamine levels below normal in mildly to moderately malnourished adolescents.
Sachs et al. [37]	2016	United States of America	Systematic review	77 articles included related to cardiac complications	N.A.	Adolescents and adults	N.A	AN	N.A	N.A	Routine echocardiography is unnecessary in AN unless symptomatic. Daily ECGs for QTc > 470 ms. Telemetry monitoring for QTc > 500 ms or sinus bradycardia <40 or junctional escape rhythm
Smythe et al. [38]	2021	N.A.	Systematic review	23 studies totalling 960 patients	N.A.	17 (mean)	15.2 kg/m^2^ (mean)	AN	N.A	N.A	Cardiac abnormalities seen in AN include reduced left ventricular mass, reduced cardiac output, increased diastolic dysfunction and increase incidence of pericardial effusions (25% of patients). Trends toward improvement with weight restoration.

RS: Refeeding Syndrome, HCR: Higher-calory refeeding, LCR: Lower-calory refeeding, %mBMI: Percent median body-mass index, AN: anorexia nervosa, AAN: atypical anorexia nervosa; %EBW: percent expected body weight; %IBW: percent ideal body weight; BN: bulimia nervosa; PD: purging disorder; ARFID: avoidant restrictive food intake disorder; UFED: Unspecified feeding and eating disorder; AN-R: anorexia nervosa restrictive subtype; AN-B/P: anorexia nervosa burge/purge subtype.

## Abbreviations

ALT	Alanine aminotransferase
AN	Anorexia Nervosa
BMI	Body-mass index
BPM	Beats per minute
CBC	Complete blood count
EBW	Expected body weight
ED	Eating disorders
EKG	Electrocardiogram
ESCAP	European Society for Child and Adolescent Psychiatry
ESPEN	European Society for Clinical Nutrition and Metabolism
GH	Growth Hormone
IBW	Ideal Body Weight
MARSIPAN	Management of Really Sick Patients with Anorexia Nervosa
mEq	Milliequivalent
PO	Per os
RS	Refeeding syndrome
SAHM	Society for Adolescent Health and Medicine
SD	Standard deviation
TSH	Thyroid stimulating hormone
WHO	World Health Organization
%mBMI	Percent median body-mass index

## Appendix A. Admission Protocol for Adolescents with Severe Anorexia Nervosa

BMI (weight in kg/square of height in m): _____ kg/m^2^

Median % BMI: (Current BMI × 100/50th centile BMI for age): ____%

*Target population: Adolescent admitted for Anorexia Nervosa and presenting with at least one criterion for increased risk of refeeding syndrome*:

- *Median % BMI < 70% or Z-score ≥ −3*
- *Oral intake <500 kcal for ≥3–4 days*
- *Weight loss of >1 kg/week for at least 2 weeks*
- *Electrolytes abnormalities before starting nutritional rehabilitation:*
- *Phosphate ≤ 1 mmol/L*
- *Potassium ≤ 3 mmol/L*
- *Magnesium ≤ 0.6 mmol/L*

**Nursing surveillance**

- Weighting: □ Daily □ Twice daily for ___ days (if clinical status worrying)
- Heart rate and blood pressure observations every □ 4 h □ 8 h for 24 h and then every _____ hours
- Temperature daily
- Orthostatic blood pressure measurement at admission
- Strict bed rest if heart rate < 40 during the night or <45 during the day
- Close observation to limit physical exercise in any form
- Continuous cardiac monitoring if heart rate < 30 BPM or QTc > 500 ms
- Inform medical team if edema
- Input and output assessment every 8 h for 48 h then revaluate
- Bedside glucose measurements before breakfast and 2 h after meals for 72 h

**Blood tests**

At admission:

- Full blood count, electrolytes, urea, creatinine, glucose, liver function tests, magnesium, phosphate
- TSH (if not done in the last 6 months)
- Others: __________________________________________________

After admission:

- Electrolytes and phosphate daily for 3 days and revaluate
- If phosphate ≤1 mmol/L, add magnesium and calcium to daily blood tests
- Others: _________________________________________________

**Other investigations**

- EKG in the first 24 h following admission (if not done in the last week)
- EKG daily if QTc > 470 ms ad normalization of prolonged QTc
- □Echocardiogram
- Bone density scan if amenorrhea for >1 year and not done previously

**Nutrition**

**Fluid intake**

- Total daily fluid intake: 20–30 mL/kg = ___________ mL/day
- Avoid IV perfusions, unless patient presents with severe hypoglycemia.

**Caloric intake**

- Start predetermined meal plan according to following recommendation, but allow 3 to 5 days for patients to entirely complete all required calories:
  **Caloric Intake**	**Initial Recommended Intake if**
  1800 kcal/day	Girls 9–12 years old or oral intake <500 kcal/day before admission
  2000 kcal/day	Girls 12–15 years old
  2200 kcal/day	Girls 15–18 years old or Boys 9–14 years old
  2500 kcal/day	Boys 14–18 years old
- If complete food refusal, critical clinical state or BMI Z score < −3 SD (approximately BMI < 12 kg/m^2^ for patients ≥12 years old):
  ○ Start NG feeding with enteric supplements with fibers at 1 kcal/mL continuous at 20 mL/h for 4 h, then increase to 40 mL/h for 4 h, then 60 mL/h and adjust according to weight gain.
  ○ Change to enteric supplement at 1.5 kcal/mL if limiting fluid intake.

**Hypoglycemia management**

- If glucose < 2.5 mmol/L and asymptomatic, offer juice and snack
- Check glucose 1 h after snack, if hypoglycemia persists, start NG feeding
- If snack refused, check glucose 1 h if continue to be asymptomatic and if hypoglycemia persists, start NG feeding.
- If severe hypoglycemia <2 mmol/L or symptomatic hypoglycemia (altered mental status or seizures):
  ○ Start Dextrose 10% bolus ____________mL (2 mL/kg) given over 15 min
  ○ Then, continue with Dextrose 10% Nacl 0/9% at __________ mL/h (3 mL/kg/h) perfusion and start simultaneously NG feeding.
  ○ Stop perfusion after two hours of NG feeding if glucose > 2.5 mmol/L.

**Medications**

- Multivitamin 1 co daily for 7 days
- Vitamin B1 (thiamine) 50 mg PO daily for 7 days
- Phosphate supplements
  ○ Consider supplementation if phosphate ≤ 1 mmol/L before starting nutritional rehabilitation or at increased risk of refeeding syndrome.
  ○ Always favor enteral route.
  ○ Verify that renal function et diuresis is normal:
    ▪ Sodium phosphate 500 mg (16 mmol) PO twice a day for 7 days (<30 kg)
    ▪ Sodium phosphate 500 mg (16 mmol) PO three times a day for 7 days (≥30 kg)
- Potassium supplements
  ○ Consider supplementation if <3 mmol/L
  ○ Verify that renal function et diuresis is normal
    ▪ Potassium chloride (KCl) 20 mmol PO three times a day for 24 h and reevaluate
- Magnesium supplements
  ○ Consider supplementation if <0.6 mmol/L
    ▪ Elementary magnesium 250 mg PO three times a day for 3 days.
